# Enhanced electromechanical coupling in piezoelectric MEMS vibration energy harvesters via strain-induced phase transition in Mn-doped bismuth ferrite epitaxial films

**DOI:** 10.1038/s41378-026-01177-5

**Published:** 2026-03-17

**Authors:** Sengsavang Aphayvong, Meika Takagi, Kira Fujihara, Yohane Fujibayashi, Norifumi Fujimura, Hidemasa Yamane, Shuichi Murakami, Takeshi Yoshimura

**Affiliations:** 1 Osaka Metropolitan University, Sakai, Osaka, 599-8531 Japan; 2 Osaka Research Institute of Industrial Science and Technology, Izumi, Osaka, 594-1157 Japan

**Keywords:** Electronic properties and materials, Electrical and electronic engineering

## Abstract

Mn-doped BiFeO_3_ (BFMO) epitaxial films grown on (100) Si wafers delivered enhanced electrical and piezoelectric properties under systematically optimized growth conditions, realized through a biaxial combinatorial sputtering method. The dielectric constant and dielectric loss of the resulting BFMO films were approximately 140 and 1%, respectively, considerably lower than those of undoped BiFeO_3_. Most notably, the effective transverse piezoelectric coefficient was –6.0 C/m^2^, the highest yet reported for this material system. According to detailed structural and electrical characterizations, the improved piezoelectric performance stems from a strain-induced phase transition from the rhombohedral to the monoclinic structure. To demonstrate this enhancement beyond the material level, the optimized films were successfully integrated into piezoelectric MEMS vibration-energy harvesters. The films demonstrated device-level performance improvements with a generalized electromechanical coupling factor $({K}^{2})$ of 0.5%, fivefold that of (100) oriented BFO films.

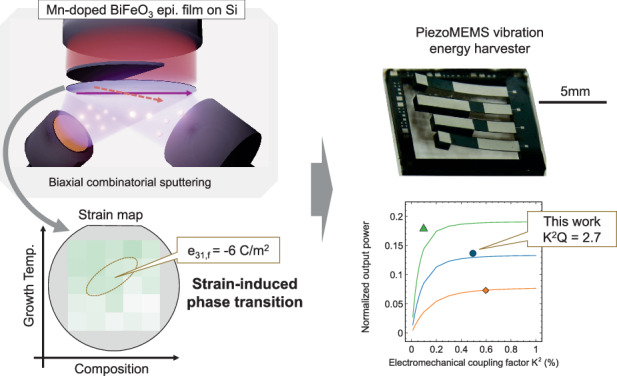

## Introduction

Since their emergence in the 1990s, microelectromechanical systems (MEMS) have revolutionized miniature sensors such as accelerometers and gyro sensors^1^. Piezoelectric thin films, emerging in the 2000s, have promised to resolve the limitations of electrostatic MEMS, enabling the precise processing of complex structures. Today, a wide variety of piezoelectric MEMS devices are available on the market, including inkjet print heads and elastic wave filters^2,3^.

Piezoelectric MEMS are broadly divided into two categories with different primary materials: Pb(Zr,Ti)O_3_ (PZT)-based and aluminum nitride (AlN)-based materials^4^. Driven by advancements in deposition technology, the quality and piezoelectric properties of PZT-based films have greatly improved over the past 30 years. The existing PZT-based films, with the effective transverse piezoelectric coefficients ${e}_{31,f}$ exceeding –20 C/m^2^, making them particularly suitable for actuator applications^4–6^. Meanwhile, the piezoelectric properties of AlN-based films have been improved by scandium incorporation. Owing to their low relative permittivity and high quality factor. AlN-based films are mainly used in resonators and sensors^7,8^.

However, given the contrasting characteristics of these two materials, keeping pace with future developments of piezoelectric MEMS devices is expected to be challenging. The latest electronic devices are integrated with artificial intelligence functions, requiring the construction of neural networks with sensors^9–11^. To meet these requirements, many sensors are arranged in arrays and must be both highly sensitive and ultra-compact. PZT-based films cannot reach such high sensitivity, whereas AlN-based films are limited by parasitic capacitance caused by their low relative permittivity.

The sensitivity characteristic of a piezoelectric thin film in sensor applications is ideally given by ${e}_{31,f}{\left({\varepsilon }_{0}{\varepsilon }_{33}\right)}^{-1}$ but is actually computed as ${e}_{31,f}^{2}{\left({\varepsilon }_{0}{\varepsilon }_{33}\right)}^{-1}$ to accommodate the finite input impedance of the detection circuit. BiFeO_3_ (BFO) is a promising piezoelectric film material because it effectively balances the piezoelectric properties, with an ${e}_{31,f}$ of –5.0 C/m^2^ and an approximate relative permittivity of 100. The multiferroic properties, domain wall conduction, and photovoltaics of BFO have been widely exploited in epitaxial films grown on oxide single-crystal substrates^12^. Recently, there has been growing interest in research aimed at practical applications, particularly in piezoelectric devices.

Prashanthi et al^13^. pioneered the use of BFO in MEMS in 2011. More recently, Liu et al. incorporated self-poled BFO films into piezoelectric micromachined ultrasound transducers^14^. The standard characteristic of PZT films is the ${e}_{31,f}$ coefficient determined from the inverse piezoelectric response, which is particularly important in MEMS applications^15,16^. In addition, ${e}_{31,f}$ plays a fundamental role in piezoelectric MEMS, as it directly affects the efficiency of electromechanical coupling in thin-film devices. Owing to recent BFO processing techniques and characterization methods, BFO materials can potentially compete with lead-based materials and are expected to be incorporated into various applications^17–21^.

In 2012, we proposed the potential usage of BFO in energy-conversion applications and demonstrated this potential in MEMS-based piezoelectric vibration-energy harvesters (MEMS-pVEHs)^22,23^. Resonant-type MEMS-pVEHs can achieve up to 90% of their theoretical maximum output at their specific resonant frequency^24^. However, to efficiently harvest environmental vibrations, which are usually time-variant, cover a broad frequency range, and are dominated by low frequencies, the ${K}^{2}{Q}_{m}$ (${K}^{2}$ = generalized mechanical coupling factor, ${Q}_{m}$= mechanical quality factor) should exceed 10, more than ten times that of resonant-type MEMS-pVEHs^25^.

To enhance the piezoelectric properties of BFO films, the present study applies an epitaxial growth scheme that precisely controls the crystallographic orientation and domain structure of the film. Domain engineering is known to enhance the piezoelectric response in both relaxor ferroelectric single crystals and epitaxial thin films^22,26–29^. Moreover, the piezoelectric performance can be further improved through strain-induced phase transitions, particularly those occurring near morphotropic phase boundaries^12,30^.

However, piezoelectric enhancement via strain-induced phase transitions has usually been demonstrated on oxide single-crystal substrates such as SrTiO_3_, LaAlO_3_, or YAlO_3_, in which lattice mismatch induces compressive strain that facilitates rhombohedral-to-tetragonal transitions^30^. For practical MEMS applications, BFO films must be grown on Si substrates, which present fundamentally different strain conditions. As the thermal expansion coefficients differ between BFO and Si, BFO films cooling from their growth temperature should experience a tensile strain rather than the compressive strain typically encountered on oxide substrates. This opposing strain state implies that knowledge obtained from oxide single-crystal studies cannot be directly applied to MEMS devices.

To address this challenge, we recently developed a sputtering-only technique for epitaxial growth of BFO films on Si substrates^31^. We also established a biaxial combinatorial sputtering approach that enables efficient exploration of the optimal growth conditions across wide parameter spaces^31,32^. Building on these technological foundations and a previous report showing that Mn doping effectively reduces the leakage current in BiFeO_3_ films^33^, we here attempt to improve the dielectric properties of Mn-doped BFO (BFMO) films while maintaining their epitaxial quality on Si substrates.

Importantly, we reveal that tensile strain on Si substrates can indeed induce strain-driven phase transitions in BFMO films. We observed a rhombohedral-to-monoclinic phase transition that substantially enhances the piezoelectric coefficients, demonstrating that strain-induced piezoelectric enhancement is achievable even under tensile-strain conditions on Si substrates. Based on these findings, we successfully epitaxially grew BFMO films on silicon-on-insulator (SOI) substrates, completing the MEMS-pVEH fabrication. Finally, we evaluated the performance of MEMS-pVEHs under simplified impulsive forces encompassing a wide range of frequency components.

## Results and discussion

### Strain-engineered phase transitions in Mn-doped BiFeO_3_ epitaxial films

**Fig. 1:**
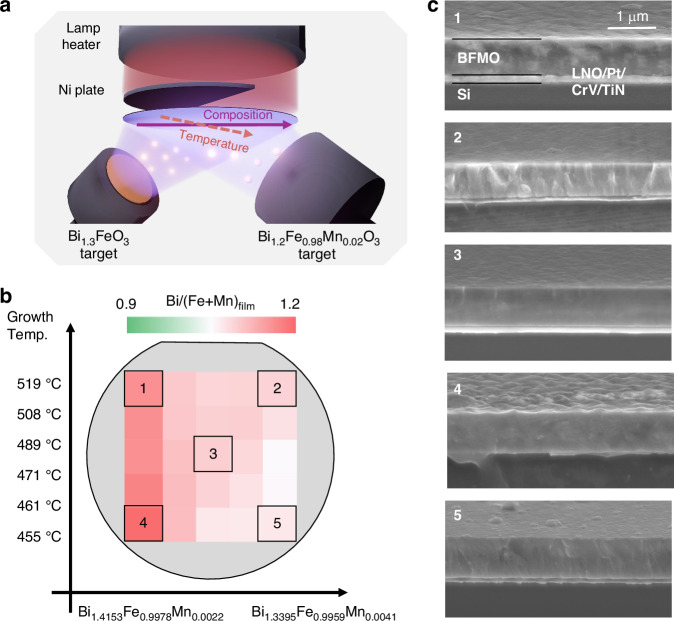
Overview of the biaxial combinatorial sputtering approach and the resulting films. **a** Schematic of the combinatorial radio frequency (RF) magnetron sputtering scheme; **b** mapping of composition ratio across a 2-inch wafer; **c** cross-sectional SEM images at various positions in the sample

The biaxial combinatorial method (Fig. 1a) enables systematic exploration of the BFMO growth conditions across wide ranges of temperature and composition. Adopting this approach, we simultaneously investigated the relationships among the growth parameters, crystalline structures, and electrical properties of the BFMO films. The biaxial method uses sintered Bi_1.3_FeO_3_ as one target and sintered Bi_1.2_Fe_0.98_Mn_0.02_O_3_ as the other, yielding varying Mn and Bi compositions across the substrate plane. A mask placed behind the substrate also creates a temperature gradient on the substrate in a direction perpendicular to the composition gradient. Figure 1b is a schematic of the 2-inch wafer mapped with the Bi-to-(Fe+Mn) composition ratios (denoted as Bi/(Fe+Mn)_film_) across 25 distinct points in the sample. The horizontal axis of the mapping is the composition ratio supplied from the targets to the substrate, determined by measuring the composition of the thin films deposited at room temperature, and the vertical axis represents the growth temperature. The Bi/(Fe+Mn)_film_ increased from 1.02 to 1.21 as the supplied Bi composition increased from 1.34 to 1.42. The Bi loss was mainly caused by Bi re-evaporation during deposition. Meanwhile, the Mn gradient varied from *x* = 1.16% to *x* = 1.34% (Fig. S1). Figure 1c shows cross-sectional scanning electron microscopy images of the sample at the positions marked 1–5 in Fig. 1b. The microstructures reveal nearly identically thick BFMO films at the five positions, despite their different deposition temperatures and compositions. All films were dense with no porous regions or structural defects. However, the films grown at lower temperatures were rough-surfaced, whereas those deposited at higher temperatures presented smoother and more uniform surfaces.

Furthermore, the results of energy dispersive X-ray spectroscopy (EDS) mapping indicate that the elemental composition is uniformly distributed across all measured positions (Fig. S2).

**Fig. 2:**
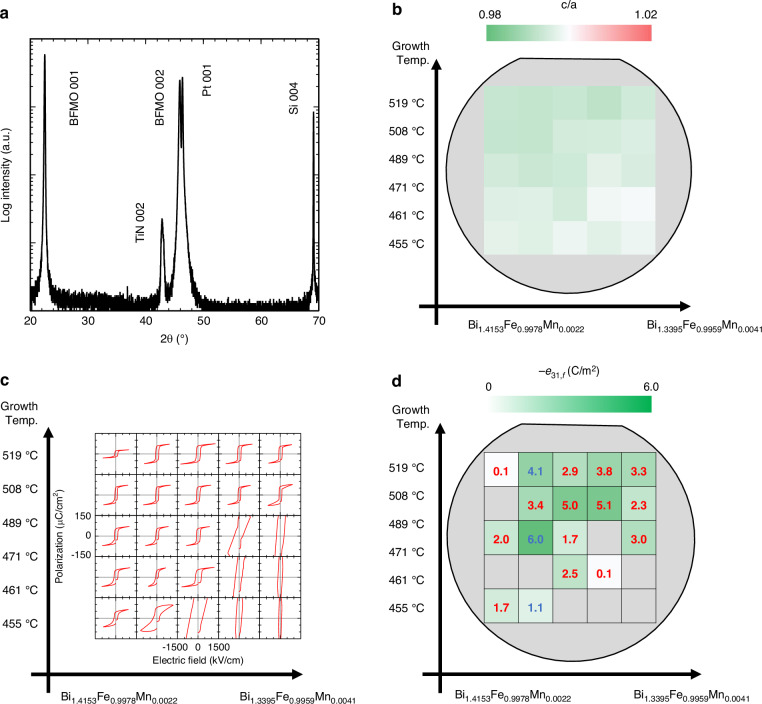
Structural and electrical property mappings of the Mn-doped BiFeO3 epitaxial films. **a** An exemplary 2*θ–ω* X-ray scan profile of the sample; mappings of **b** lattice constant ratios, **c** *P–E* hysteresis loops, and **d** effective transverse piezoelectric coefficient at various positions in the sample

Figure 2a shows the X-ray diffraction 2θ–ω profile obtained at the center of the wafer. All strong diffraction peaks from the BFMO (00 *l*) are observed, with no evidence of misoriented growth or secondary phase formation. Similar diffraction patterns were obtained across the entire wafer surface (data not shown). In addition, φ-scan measurements confirmed the epitaxial growth of BFMO films on the (100) Si substrate, with a cube-on-cube relationship throughout the films (Fig. S3).

To analyze the detailed crystal structures, reciprocal space mapping (RSM) of the BFMO ($\bar{2}$03) diffraction was measured across 25 distinct points on the sample (see Fig. S4). Bulk BFO is known to possess a rhombohedral structure (lattice constant: 3.96 Å, *β* = 89.4°). The two distinct diffraction peaks in the RSM indicate the retention of rhombohedral-like distortion in the BFMO films. Importantly, this rhombohedral structure was consistently observed across the wafer, confirming uniform epitaxial growth under different local growth conditions.

However, the peak positions systematically varied with composition and growth temperature, clearly shifting from those of bulk BFO. To quantify these structural changes, we determined the out-of-plane lattice parameter *c* and in-plane lattice parameter *a* from the peak positions and calculated the *c*/*a* ratios. As shown in Fig. 2b, the *c*/*a* ratios were mainly below one, and a clear trend is observed: at substrate temperatures around 420 °C, the c/a ratio approaches one, but gradually decreases as the substrate temperature increases. This structural evolution indicates a rhombohedral (*R3c*)-to-monoclinic (*M*_*B*_) phase transition in the BFO film as the temperature increases, which can be explained by tensile strain arising from the thermal expansion coefficient mismatch between BFMO and the Si substrate (Si: $\sim 3\times {10}^{-6}$ deg^–1^, BFO: $\sim 1\times {10}^{-5}$ deg^–1^). The observed transition is consistent with previous reports under tensile-strain conditions^34^. A key observation is the higher stability of the *R3c* structure in the Mn-doped film than in undoped BFO epitaxial films on Si^31^. This stabilization enables the coexistence of different structural phases across the wafer, effectively creating a strain-induced phase-boundary distribution within the sample and allowing systematic investigation of the relationship between crystal structure and piezoelectric properties.

Figure 2c shows the polarization–electric field (*P–E*) hysteresis loops across the sample, measured at 100 kHz. The *P–E* loops were rectangular except in regions with low Bi composition. Given that undoped BFO films exhibit ferroelectricity even when grown below 450 °C^31^, doping appears to raise the optimal growth temperature by 50 °C from that of undoped BFO films. Furthermore, the doped BFMO films grown under optimal conditions show low leakage current and small dielectric dispersion (Fig. S5). The remarkably low relative permittivity (~140) and dielectric loss (~0.01) at frequencies below 1 kHz are particularly valuable in MEMS applications. While the number of measurable samples was limited because the wafer had to be cut into small pieces, Fig. 2d summarizes the measured effective transverse piezoelectric coefficients ${e}_{31,f}$ (Fig. S6). Lower ${e}_{31,f}$ values were observed due to film degradation commonly observed at wafer edges. This degradation, characterized by increased particle contamination, results in lower breakdown voltage, preventing adequate poling treatment. Therefore, the observed distribution reflects both the intrinsic improvement in piezoelectric properties within the high-quality central region and the quality-related limitations in the edge regions. The ${e}_{31,f}$ coefficient increased where the BFMO crystal structure transitioned from the *R3c* to the *M*_*B*_ phase. The maximum piezoelectric coefficient was –6.0 C/m^2^ in the stoichiometric region.

**Fig. 3:**
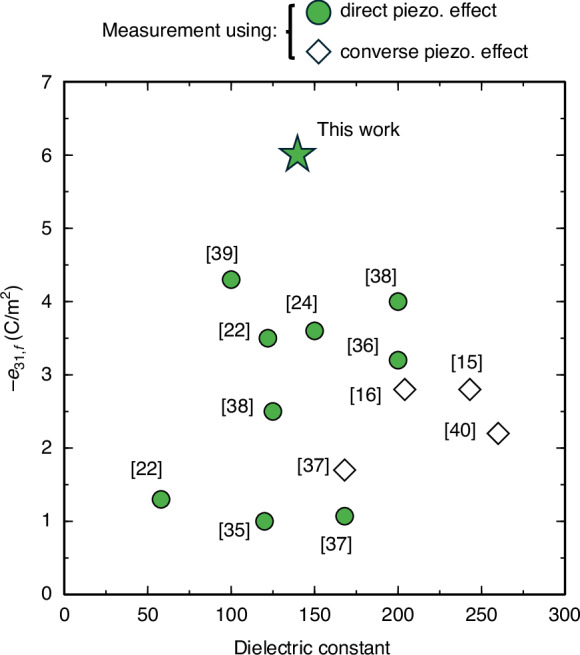
Dependence of ${e}_{31,f}$ on dielectric constant in the present work and in literature-reported BiFeO_3_-based films, characterized by direct (filled symbols) and converse (open symbols) piezoelectric effects. The numbers are the references to the publications

For comparison, Fig. 3 summarizes the ${e}_{31,f}$ values of the BFO films published to date^15,16,22,24,35–40^. Owing to extrinsic piezoelectric effects, the converse piezoelectric effect generally yields larger measured ${e}_{31,f}$ values than the direct piezoelectric effect^41^ (unfilled diamonds vs. green filled symbols in Fig. 3). The (100) BFMO epitaxial film grown on Si substrate in this work achieved the highest ${e}_{31,f}$ among the currently reported BFO-based thin films. Depending on its concentration and valence state, the Mn dopant induces a strain-driven rhombohedral-to-tetragonal or rhombohedral-to-orthorhombic phase transition in BFO^42,43^. However, most studies have shown only that Mn doping improves the ferroelectricity, ferromagnetic properties, and optical properties of BFO thin films^44,45^.

The present study demonstrates that Mn doping alters the phase stability of BFO films on Si substrates. Undoped BFO films immediately adopt the *M*_*B*_ phase under the tensile strain imposed by thermal expansion mismatch, preventing any phase-transition behavior with increasing tensile strain. Mn doping modifies the critical strain threshold of the *R3c*-to-*M*_*B*_ transition, which allows the *R3c* phase to exist at moderate tensile strain levels. The combinatorial temperature gradient creates a systematically variable tensile strain across the wafer, enabling us to traverse the modified phase boundary and observe the *R3c*-to-*M*_*B*_ transition that would be impossible in undoped BFO. The enhanced piezoelectric properties arise from this controlled phase boundary-region.

### Fabrication and harmonic response characterization of the MEMS energy harvester

**Fig. 4:**
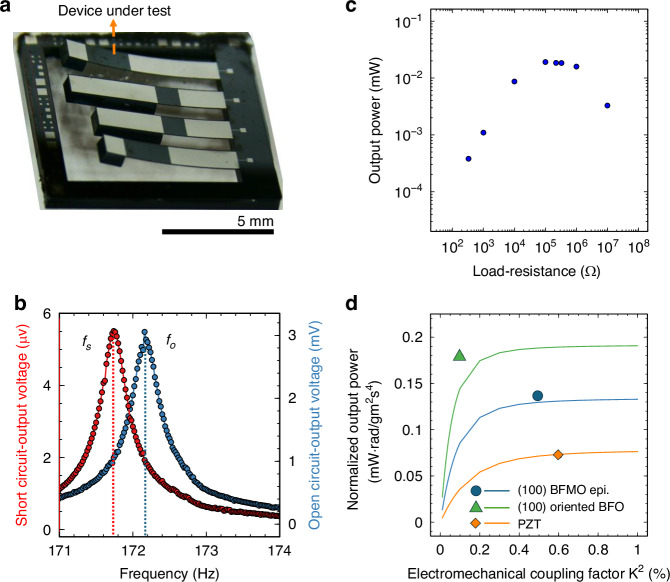
Performance characterization of MEMS devices under harmonic vibration. **a** Photograph of the fabricated MEMS-pVEHs; **b** resonance curve under the short- and open- circuit condition; output power dependences of **c** load resistance, and **d** generalized mechanical coupling factors ${K}^{2}$, where the mechanical quality factors ${Q}_{m}$ = 313, 536, and 766 for the PZT, (100) oriented BFO, and (100) BFMO epitaxial films, respectively

Based on the results described above, MEMS-pVEHs were fabricated using epitaxially grown BFMO films. The BFMO films were uniformly deposited on a (30 × 30 mm^2^) SOI substrate, and the devices were fabricated through a conventional MEMS process. Figure 4a is an optical micrograph of the cantilever-structured MEMS-pVEH. The cantilevers, which bend upward under tensile stress from the SOI substrate, are 1.0 mm wide, 7.0 mm long, and 20 μm thick. The silicon proof mass of the test device was approximately 1.4 mg.

The performances of the MEMS-pVEH were characterized under steady excitation by applying sinusoidal vibration to the harvester. The measured vibration-frequency dependencies of the output voltage under short (*R* = 330 Ω) and open (*R* = 10 MΩ) circuit equivalent conditions are shown in Fig. 4b. Setting the input acceleration to 0.005 m/s^2^, ${K}^{2}$ was calculated as^46^

1 $${K}^{2}=\frac{{\omega }_{{open}}^{2}-{\omega }_{{short}}^{2}}{{\omega }_{{short}}^{2}}$$

where ${\omega }_{{short}}$ and ${\omega }_{{open}}$ are the angular resonance frequencies under short and open circuit conditions, respectively. The mechanical quality factor ${Q}_{m}$ was determined from the resonance curve of the output voltage under the short circuit condition using the half-bandwidth method as follows:

2 $${Q}_{m}=\frac{{\omega }_{{short}}}{{\omega }_{2}-{\omega }_{1}}$$

where ${\omega }_{2}$ and ${\omega }_{1}$ are the angular resonance frequencies at which the maximum voltage is $1/\sqrt{2}$ of the maximum voltage. From Eqs. (1) and (2), ${K}^{2}$ and ${Q}_{m}$ were determined as 0.5% and 536, respectively. The highly important figure of merit of pVEH, the product ${K}^{2}{Q}_{m}$, was 2.7, almost three times higher than that of an undoped (100) oriented BFO film. Furthermore, when the Si layer is much thicker than the piezoelectric film, ${K}^{2}$ can be approximated as

3 $${K}^{2}\approx \frac{{h}_{p}{Y}_{p}}{{h}_{s}{Y}_{s}}{k}_{31,f}^{2}$$

4 $${k}_{31,\,f}^{2}=\frac{{\left(1-v\right)}^{2}{e}_{31,f}^{2}}{{Y}_{p}{\varepsilon }_{0}{\varepsilon }_{33}}$$

where ${h}_{p}$ and ${h}_{s}$ are the thicknesses of the piezoelectric film and Si layer, respectively, ${Y}_{s}$ and ${Y}_{p}$ are the Young’s modulus of the Si layer and piezoelectric film, respectively, ${k}_{31,f}^{2}$ is the electromechanical coupling factor of the piezoelectric film in 31 mode, and $v$ is the Poisson’s ratio of the substrate. Setting ${h}_{p}$ = 800 nm, ${h}_{s}$ = 20 μm, and ${Y}_{s}$ = 196 GPa, the ${e}_{31,f}$ coefficient was calculated as –5.1 C/m^2^. This value is slightly lower than the –6.0 C/m^2^ measured on the as-deposited BFMO film, which is due to the damage of the piezoelectric film during the MEMS process^24,46^. Further optimization of the MEMS fabrication process, particularly minimizing plasma damage, will be necessary to fully preserve the intrinsic piezoelectric performance of the BFMO film. Moreover, piezoelectric characterization of additional MEMS devices fabricated from the same batch on the same substrate revealed similar ferroelectric properties and similar ${e}_{31,f}$ coefficient (Fig. S7). This consistency confirms that the BFMO films were uniformly fabricated across the entire substrate, demonstrating the feasibility of batch fabrication with reliable piezoelectric performance.

Although the load resistance for impedance matching is fixed at 100 kΩ, the matching conditions are broadened, thanks to the high ${K}^{2}{Q}_{m}$. The output power $P$ of a pVEH with linear resonance under the impedance-matching and resonant-frequency conditions is given as follows^47^:

5 $$P=\frac{m{A}^{2}{Q}_{m}}{4{\omega }_{r}\left(1+\sqrt{1+\frac{1}{{K}^{4}{Q}_{m}^{2}}}\right)}$$

where *A* is the input acceleration, and *ω*_*r*_ is the angular resonance frequency. Figure 4d compares the theoretical value obtained by Eq. (5) with the experimental results, including our previous results on the (100) oriented BiFeO_3_ film (m: 3.0 mg, ${K}^{2}$: 0.1%, ${Q}_{m}$: 766), and a PZT film (m: 4.2 mg, ${K}^{2}$: 0.6%, ${Q}_{m}$: 313)^25^. Owing to their higher ${Q}_{m}$, the MEMS-pVEHs employing the BFO and BFMO films delivered higher normalized $P$ than the PZT film. The (100) BFMO epitaxial film, (100) oriented BFO film, and PZT yielded ${K}^{2}{Q}_{m}$ values of 2.7, 0.8, and 1.9, respectively, and the ratios of the output power to the theoretical maximum were calculated as 98%, 76%, and 96%, respectively. Although the ${K}^{2}{Q}_{m}$ value is rarely mentioned in MEMS-pVEH studies, we can assume that the fabricated MEMS-pVEH using the (100) BFMO epitaxial film is the highest-${K}^{2}{Q}_{m}$ device to date.

### Broadband energy harvesting under impulsive excitation

**Fig. 5:**
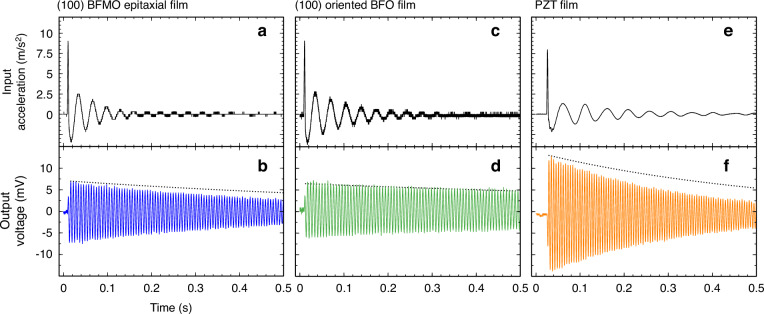
Dynamic waveforms of the MEMS-pVEH devices. Input impulsive waveforms and output voltage waveforms of the BFMO device (**a** and **b**, respectively), the non-doped BFO device (**c** and **d**, respectively), and the PZT device (**e** and **f**, respectively)

As pVEHs with high ${K}^{2}{Q}_{m}$ are demanded for power generation under non-resonant conditions, the output power of the MEMS-pVEH was investigated under an impulsive force. Figure 5 illustrates the input impulsive forces and corresponding output voltage waveforms of the MEMS-pVEH, along with those of (100) oriented BFO- and PZT-based samples for comparison. After applying the impulsive force, the output response exhibited free-damped oscillations with an output voltage proportional to the proof mass and ${K}^{2}{Q}_{m}$. Interestingly, the BFMO sample outputted a maximum voltage equivalent to that of the (100) oriented BFO sample despite its smaller proof mass (one-half the proof mass of BFO), due to high ${K}^{2}{Q}_{m}$. Furthermore, the oscillations in the output voltage were damped more slowly in the BFO sample than in the other samples. The black dotted lines in the bottom panels of Fig. 5 display the mechanical damping curves of the oscillators. The increase in damping rate with ${K}^{2}$ (Fig. 5b, f) is attributable to electrical damping associated with the piezoelectric effect. The durations over which the output voltage declined from its peak to 10% of the maximum were measured as 1.2, 3.4, and 0.7 s in the MEMS-pVEHs using the BFMO, BFO, and PZT films, respectively, yielding ${K}^{2}$ values of 0.5%, 0.1%, and 0.7%, respectively.

**Fig. 6:**
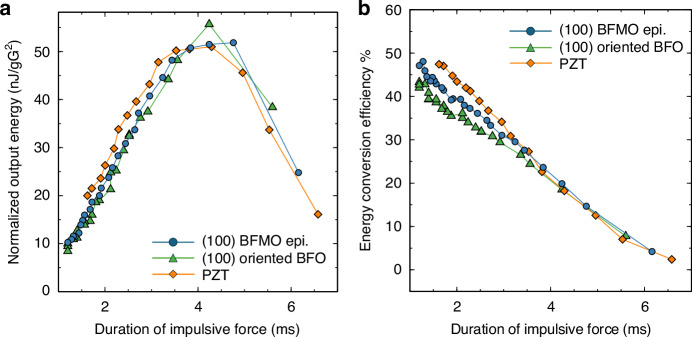
Energy harvesting performance of MEMS devices under impulsive excitation. **a** Normalized output energy and **b** energy-conversion efficiency as functions of input impulse duration

Figure 6a plots the output-energy dependence on impulse duration for the three MEMS-pVEH devices. The performance of the BFO-based MEMS-pVEH approached that of the PZT-based device. The output-energy-conversion efficiency was calculated as

6 $${E}_{{out}}=\frac{1}{m{A}^{2}}{\int }_{\!0}^{t}\frac{({V}_{p}{\left(t\right))}^{2}}{{R}_{L}}{dt}$$

7 $${E}_{{in}}=\frac{1}{2}m{\left({\int }_{\!0}^{{t}_{d}}y\left(t\right){dt}\right)}^{2}$$

where $t$ is the measurement duration after applying the impulsive force and ${t}_{d}$ is the duration of the impulsive force. As shown in Fig. 6b, the energy-conversion performances of the three MEMS-pVEHs with varying parameters were very similar. The output voltage under an impulsive force was integrated over time $t=2{\rm{s}}$, to obtain the normalized output energy. Under sinusoidal vibration, the BFO showed the highest power output due to its high ${Q}_{m}$ but when subjected to an impulsive force, all three devices produced nearly identical energy outputs. For frequency-up conversion, which effectively harvests energy from random low-frequency vibrations in the environment, a high output voltage and quick damping are beneficial because they allow frequent applications of successive impulsive forces. For example, the output power of the BFMO decayed to 10% of its maximum at 1.2 s, nearly three times shorter than the decay time of the MEMS-pVEH with the BFO film (3.4 s). Therefore, the BFMO is ready for the next impulse while the BFO remains in the damping process. As the impulsive force can be applied three times more frequently to the BFMO than to the BFO, the BFMO is better suited to frequency-up conversion.

## Conclusion

The fabrication conditions of BFMO films were systematically optimized using a biaxial combinatorial RF sputtering method. Although the processing range of BFMO was reduced from that of non-doped (100) BFO films, the electrical properties improved, and the leakage current decreased. The small amount of Mn doped in the (100) BFMO film conferred a high piezoelectric coefficient (–6.0 C/m^2^). Next, MEMS-pVEHs were fabricated by depositing BFMO film on a SOI substrate. The MEMS-pVEH fabricated with an 800-nm-thick BFMO film achieved an ${e}_{31,f}$ coefficient of –5.1 C/m^2^, the generalized electromechanical coupling factor ${K}^{2}$ of 0.5%, and a mechanical quality factor ${Q}_{m}{\rm{of}}\,536$, comparable to that of PZT film. The ${K}^{2}Q$ of the MEMS-pVEH was 2.7, indicating a generated power above 90% of the theoretical maximum output. We also compared the output powers of MEMS-pVEHs fabricated from various piezoelectric films under sinusoidal vibrations. The (100) oriented BFO and BFMO films produced higher output power than the PZT film, owing to their high piezoelectric coefficients, although the energy outputs under a single impulsive force were similar across all films. High output voltages with quick damping are desired for frequency up-conversion because they enhance the efficiency and allow frequent applications of impulsive forces. The MEMS-pVEH based on the (100) BFMO film offers distinct advantages for this purpose.

## Materials and methods

All bottom electrode layers were uniformly fabricated by sputtering on a (100) Si wafer. TiN was deposited as the first epitaxial buffer layer on the Si substrate, followed by Pt as the oxygen barrier layer, and LaNiO_3_ (LNO) is the seed layer. The BFMO epitaxial film was then deposited using the biaxial combinatorial sputtering method. The deposition details are reported elsewhere^31,32^. The temperature gradient was measured using black painted Si substrate because mirror-finished Si wafers have low emissivity, making it difficult to accurately measure their temperature with infrared. A thermographic image was obtained using an infrared camera (Xi 400, Optris GmbH, Berlin, Germany) through an infrared-transparent BaF₂ window. The temperature measured by the infrared camera was calibrated using a reference sample equipped with a thermocouple. The supplying compositions from the targets were measured by measuring two points, one near and one far from the BFMO target deposited at room temperature, using the inductively coupled plasma atomic emission spectrometry method. The crystalline structures of the films were determined from X-ray diffraction patterns, 2*θ–ω* scans, *φ* scans, and reciprocal space mapping (X’pert-MRD, Phillips, Almelo, Netherlands) using Cu-K_α1_ radiation. The compositions of the films were determined using an electron probe microanalyzer (JXA-8539F, JEOL, Tokyo, Japan).

The electrical properties of the films were determined from the polarization–electric field characteristics measured using a ferroelectric tester (Precision Multiferroic II, RADIANT Technologies, Inc., New Mexico, USA). ${e}_{31,f}$ coefficients were measured based on the direct piezoelectric response, the wafer flexure method^48,49^, and the cantilevered bending method^50^.

**Fig. 7:**
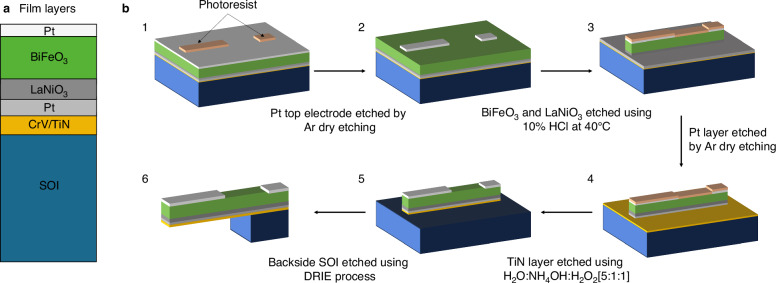
Device structure and fabrication process of the piezoelectric MEMS vibration energy harvesters. **a** Sample structure and **b** fabrication process of the MEMS-pVEHs

The MEMS-pVEHs were fabricated on SOI substrates. To improve the leakage current properties of the BFO film, the thickness of the LNO seed layer was raised to 150 nm. The MEMS-pVEHs were fabricated at a stoichiometric composition by applying the same amount of power to the target during deposition. After growing a uniform 800-nm-thick BFMO film, the 60-nm-thick top electrode was deposited. The top electrodes were dry etched using Ar, and both BFMO and LNO were etched with 10% HCl at 40 °C. Subsequently, the underlying Pt bottom electrode was then dry etched using Ar, and the TiN buffer layer was etched with a mixed acid, specifically, a 5:1:1 volumetric ratio of H_2_O, NH_4_OH, and H_2_O_2_. Next, the Si layer was etched via the deep reactive-ion etching process (see Fig. 7).

The cantilevers were designed to respond to common environmental vibration sources with typical frequencies below 200 Hz. The structures were designed and optimized to an appropriate resonance frequency and strain distribution using finite element method simulations. A Pt layer was deposited at the tip of the cantilever for precise displacement measurements using a laser vibrometer. Finally, as the device is intended for low-frequency vibration-energy harvesting, a proof mass was added to its cantilever tip to reduce the resonance frequency.

For electromechanical characterization, the devices were mounted on a standard universal board. The electrodes were wire-bonded and connected to conductive copper tape fixed to the universal board for electrical contact. The MEMS-pVEH were excited with sinusoidal vibrations and impulsive forces using a shaker (PET-1, IMV Corp., Osaka, Japan). The applied acceleration was measured using an accelerometer and a lock-in amplifier (LI5640, NF Corp., Yokohama, Japan). Load resistors were connected to the MEMS-pVEH for measuring the generated electrical power. The output voltage across the load resistors was measured using the lock-in amplifier, and the response waveforms were observed on an oscilloscope.

## Supplementary information

Supplemental Material

## Data Availability

The data supporting the study findings are available from the corresponding author upon reasonable request.
