# Supplementary material for: Enhanced electromechanical coupling in piezoelectric MEMS vibration energy harvesters via strain-induced phase transition in Mn-doped bismuth ferrite epitaxial films
Source: Microsyst Nanoeng. 2026 Mar 17;12:90. doi: 10.1038/s41378-026-01177-5 (PMC12993028; doi:10.1038/s41378-026-01177-5)
Supplement: Supplementary file 1 — Supplemental Material [file 41378_2026_1177_MOESM1_ESM.docx]

Enhanced Electromechanical Coupling in Piezoelectric MEMS Vibration Energy Harvesters via Strain-induced Phase Transition in Mn-doped Bismuth Ferrite Epitaxial Films

Sengsavang Aphayvong^a^, Meika Takagi^a^, Kira Fujihara^a^, Yohane Fujibayashi^a^, Shuichi Murakami^b^, Hidemasa Yamane^b^, Norifumi Fujimura^a^, Takeshi Yoshimura^a^ *

*^a^ Osaka Metropolitan University, Sakai, Osaka 599-8531, Japan*

*^b^ Osaka Research Institute of Industrial Science and Technology, Izumi, Osaka 594-1157, Japan*

**Corresponding Author:** Takeshi Yoshimura, Email: yoshimura@omu.ac.jp

**Supporting Figures**

Figure S1. Mn composition at various positions in the wafer.

Figure S2 Cross-sectional scanning electron microscopy (SEM) images and energy dispersive X-ray spectroscopy (EDS) mappings at various positions in the sample.

Figure S3 Examples of ϕ-scan profiles of the sample.

Figure S4. Reciprocal space mappings of the ($\bar{2}$03) BFMO diffraction pattern, obtained at 25 distinct points within a Mn-doped BFO film grown via combinatorial radio-frequency magnetron sputtering.

Figure S5. (a) Leakage current and (b) dielectric properties of the BFMO film at various positions in the wafer.

Figure S6. Example showing the dependence of the $e_{31,f}$ piezoelectric coefficient on the poled electric-field measurements.

Figure S7. (a) Photograph of the uniformly fabricated BFMO on SOI substrate; (b) ferroelectric properties at various positions in the film; (c) Resonance curves under short open circuit condition for determining $K^{2}$ and $e_{31,f}$ of the device in test I (mentioned in the manuscript) and test II, respectively.
